# Editorial: Calcitonin Gene-Related Peptide: Novel Biology and Treatments

**DOI:** 10.3389/fphys.2022.964568

**Published:** 2022-07-19

**Authors:** Susan D. Brain, Andrew F. Russo, Debbie L. Hay

**Affiliations:** ^1^ Section of Vascular Biology and Inflammation, School of Cardiovascular Medicine and Research, BHF Centre of Excellence, King’s College London, London, United Kingdom; ^2^ Department of Molecular Physiology and Biophysics, University of Iowa, Iowa City, IA, United States; ^3^ Department of Neurology, University of Iowa, Iowa City, IA, United States; ^4^ Center for the Prevention and Treatment of Visual Loss, Veterans Administration Health Center, Iowa City, IA, United States; ^5^ Department of Pharmacology and Toxicology, University of Otago, Dunedin, New Zealand

**Keywords:** CGRP–Calcitonin Gene-Related Peptide, migraine, adverse (side) effects, receptor, cardiovascular disease, antibodies and antagonists, receptor (biochemistry)

Calcitonin gene-related peptide (CGRP) is a 37 amino acid neuropeptide originally discovered in 1982 as a product of alternative splicing of the calcitonin gene. It was realised over the next few years that CGRP is primarily localised to sensory nerves and is a potent microvascular vasodilator. During these times evidence started to emerge that CGRP may be of functional importance in the cerebral circulation and consequently of potential relevance to migraine. By comparison it took a surprisingly long time to decipher the unique structure of the CGRP receptor family. This was finally achieved in 1998. So, by the start of the 21^st^ century, CGRP was known to have a range of potent biological activities and to be of potential relevance to the treatment of migraine (Russell et al., 2014). Since then, much has happened in terms of new science and drug discovery. CGRP and CGRP receptor antibodies have been used to benefit those living with migraine for several years and now small molecule CGRP receptor antagonists are beginning to play a role in the therapeutic landscape for migraine. This Frontiers theme showcases and overviews the broader biological discoveries in recent years. Additionally, we report how use of the CGRP antibodies and antagonists for migraine has allowed us to further decipher the wider biological importance of CGRP.

It is perhaps surprising that the expression and localization of these peptides and their receptors are still under study. However, it is only now becoming widely acknowledged that an amylin receptor (calcitonin receptor [CTR]-receptor activity-modifying protein 1 [RAMP1]; AMY_1_), in addition to the CGRP receptor (calcitonin receptor-like receptor [CLR]-RAMP1), is potently activated by CGRP and therefore, knowledge of their expression is of importance in trigeminal ganglia neurons. Rees et al. show in this theme that unlike CLR, CTR is co-localised in trigeminal C- rather than A-fibres, including in human. Thus, CTR and CGRP are co-localised in a site where CGRP may autoregulate its own expression by a positive feedback loop to influence migraine and other cerebral conditions. Indeed, following along this line Edvinsson et al. discuss signalling within the trigeminovascular system and how important this is in our search for novel ways to treat migraine. Moreover, a review by Balcziak and Russo, emphasises the close link that CGRP has with dural structures including immune cells and genes and discusses their potential to be involved in migraine pathology.

Today, knowledge is continuing to increase concerning the Class B G protein-coupled receptor CGRP receptor family and ways in which the receptors may be targeted in the future. A study by Pearce et al. uses cutting edge technologies to demonstrate biased CLR-RAMP1 receptor signalling. They investigated receptor desensitisation and the downstream pathways to reveal the role of arrestins and associated kinases. There are a number of CGRP blocking antibodies and CGRP receptor antagonists available for the treatment of migraine. Current antagonists are either antibodies or small molecules but peptide antagonists offer a potential alternative. In this theme there is an article by Jamaluddin et al. on their approach to lipidate peptide antagonists to increase their half-life. The results show that whilst the study is at an early stage, there are positive aspects for applying lipidation to create novel peptide antagonists.

The precise mechanisms *via* which CGRP influences migraine remain unclear. However, the vasodilator effect of CGRP on cranial arteries and link with migraine is established. One mechanism *via* which CGRP dilates is *via* K_ATP_ channels. Coskun et al. shows that the K_ATP_ channel antagonist glibencamide had no effect on CGRP-induced headache or CGRP-induced vasodilation. Whilst interesting, there are questions concerning the dose and selectivity, or whether the CGRP-induced K_ATP_ activation is observed in humans. On the other hand, it is striking that whilst CGRP is a potent vasodilator, the use of CGRP antibodies and antagonists for migraine has met with relatively few adverse effects on the cardiovascular system. However, it should be noted that at least one CGRP blocking antibody is linked to an FDA hypertension warning. This could mean that insufficient CGRP is released in humans. An interesting manuscript by Skaria and Vogel suggests that CGRP, known to be released in exercising humans, may be influential in mediating cardiovascular protective effects in both physiological and pathological situations. Furthermore, CGRP antibodies and antagonists, were suggested by these authors to remove the long-term benefit of exercise. Their original data was obtained in exercising WT and CGRP knockout mice. This is complemented by reviews presented in the theme from Argunhan and Brain and the Kumar et al. that emphasise the potential importance of CGRP agonists as therapeutic agents to treat cardiovascular disease. CGRP is also involved as a protective factor in immune disease such as discussed in this theme by Mariotton et al. from Ganor’s lab.

One of the most interesting aspects of CGRP biology is learning about its pathophysiological importance through administration of the CGRP blockers in migraine. Whilst the low incidence of side effects has been acknowledged worldwide, a surprising side effect in some has been constipation. In this issue Holzer and Holzer-Petsche, uses their long-standing experience in this research area to attempt to unravel the mechanisms involved. It is suggested that blocking the role of CGRP in peristalsis and secretion in the intestine underlies this side-effect of CGRP-targeted therapeutics.

The collective works presented in this research theme allow a better understanding of CGRP, both with respect to biological activities and as a druggable target. There are still many answers to find and research on CGRP remains at a high level of interest today.
